# REDOX IMAGING OF THE p53-DEPENDENT MITOCHONDRIAL REDOX STATE IN COLON CANCER *EX VIVO*

**DOI:** 10.1142/S1793545813500168

**Published:** 2013-06-11

**Authors:** HE N. XU, MIN FENG, LILY MOON, NATHAN DOLLOFF, WAFIK EL-DEIRY, LIN Z. LI

**Affiliations:** * Department of Radiology, University of Pennsylvania, Philadelphia, PA, USA; † Britton Chance Laboratory of Redox Imaging, Johnson Research Foundation, Department of Biochemistry and Biophysics, Perelman School of Medicine, University of Pennsylvania, Philadelphia, PA, USA; ‡ Department of Medicine, Penn State Hershey Medical Center and Penn State College of Medicine, Hershey, PA, USA; § Hematology/Oncology, Penn State Hershey Medical Center, Hershey, PA, USA; ¶ Abramson Cancer Center, University of Pennsylvania, Philadelphia, PA, USA; || Institute of Translational Medicine and Therapeutics, University of Pennsylvania, Philadelphia, PA, USA

**Keywords:** NADH, flavoprotein, intratumor heterogeneity, HCT116, p53 null, necrotic

## Abstract

The mitochondrial redox state and its heterogeneity of colon cancer at tissue level have not been previously reported. Nor has how p53 regulates mitochondrial respiration been measured at (deep) tissue level, presumably due to the unavailability of the technology that has sufficient spatial resolution and tissue penetration depth. Our prior work demonstrated that the mitochondrial redox state and its intratumor heterogeneity is associated with cancer aggressiveness in human melanoma and breast cancer in mouse models, with the more metastatic tumors exhibiting localized regions of more oxidized redox state. Using the Chance redox scanner with an in-plane spatial resolution of 200 *μ*m, we imaged the mitochondrial redox state of the wild-type p53 colon tumors (HCT116 p53 *wt*) and the p53-deleted colon tumors (HCT116 p53^−/−^) by collecting the fluorescence signals of nicotinamide adenine dinucleotide (NADH) and oxidized flavoproteins [Fp, including flavin adenine dinucleotide (FAD)] from the mouse xenografts snap-frozen at low temperature. Our results show that: (1) both tumor lines have significant degree of intratumor heterogeneity of the redox state, typically exhibiting a distinct bi-modal distribution that either correlates with the spatial core–rim pattern or the “hot/cold” oxidation-reduction patches; (2) the p53^−/−^ group is significantly more heterogeneous in the mitochondrial redox state and has a more oxidized tumor core compared to the p53 *wt* group when the tumor sizes of the two groups are matched; (3) the tumor size dependence of the redox indices (such as Fp and Fp redox ratio) is significant in the p53^−/−^ group with the larger ones being more oxidized and more heterogeneous in their redox state, particularly more oxidized in the tumor central regions; (4) the H&E staining images of tumor sections grossly correlate with the redox images. The present work is the first to reveal at the submillimeter scale the intratumor heterogeneity pattern of the mitochondrial redox state in colon cancer and the first to indicate that at tissue level the mitochondrial redox state is p53 dependent. The findings should assist in our understanding on colon cancer pathology and developing new imaging biomarkers for clinical applications.

## 1. Introduction

Cancer metabolism has received increasing research interest in recent years. Metabolic alteration, a hallmark of cancer, allows cancer cells to adapt to their needs for rapid bioenergetics, increased biogenesis of macromolecules and maintenance of the redox balance.^1^ p53, as a tumor suppressor gene, regulates many metabolic pathways including glycolysis and mitochondrial respiration in tumors.^2^ p53 plays a central role in mitochondrial oxygen utilization, reactive oxygen species generation, and disposition.^3,4^

Previously we demonstrated that tumor meta-static potential was associated with the mitochondrial redox state and its intratumor heterogeneity in both melanoma and breast cancer mouse xenografts models.^5–7^ We showed that the more metastatic tumors have more oxidized mitochondrial redox state in the localized regions and the quantitative redox indices can grade tumor aggressiveness. We also discovered that mitochondrial redox state alteration was linked to the activation of the PI3K/ Akt pathway due to PTEN deletion and cancer transformation.^8^ As p53 influences the PI3K/Akt pathway through activating PTEN, it promotes mitochondrial bioenergetics by activating the expression of synthesis of cytochrome c oxidase 2 (SCO2), and inhibits the glycolysis pathway,^3,4,9^ we hypothesize that p53 status may also affect the mitochondrial redox state. To test this hypothesis, we employed the Chance redox scanner to image the fluorescence of nicotinamide adenine dinucleotide (NADH) and flavoprotein (Fp) with 3D sub-millimeter resolution in two colon cancer lines xenografted in mice, one being p53 *wt* and the other p53^−/−^. Our findings indicate that the tumor mitochondrial redox state is dependent on p53 status.

## 2. Materials and Methods

### 2.1. Tumor growth

Human colon cancer lines HCT116 p53 *wt* and HCT116 p53^−/−^ were cultured and propagated in McCoy’s 5A medium supplemented with 10% fetal bovine serum and 100 unit/ml penicillin and 100 *μ*g/ ml streptomycin. Equal amount (1–2.5 millions) of each line in Matrigel (50/50%) was inoculated subcutaneously to the left and right upper thighs of the same athymic nude mouse (US National Cancer Institute, NCr-nu/nu), respectively. Tumor volume was measured weekly using a vernier caliper. In about 3–4 weeks, the mice under anesthesia were sacrificed using the snap-freezing protocol with liquid nitrogen. The snap-freezing protocol maintains the tissue redox state the same as the *in vivo* condition. The tumors were then harvested and embedded for redox scanning in a liquid nitrogen chamber in the same way as described previously^6,10^ with NADH and flavin adenine dinucleotide (FAD) reference standards of known concentrations (100 *μ*M and 525 *μ*M, respectively in pH 7 Tris-HCL buffer) placed adjacent to the tumor samples.

### 2.2. Redox scanning and data analysis

The Chance redox scanner^11,12^ was employed to scan the tumors section by section spacing 400 *μ*m. The starting section is immediately beneath the skin. 3–5 sections were scanned for each tumor. The optical filters are 365BP26 (Ex) and 455DF70 (Em) for the NADH channel and 440DF20 (Ex) and 515DF30 (Em) for the Fp channel. The NADH and Fp signals from both the tissue and the reference standards were collected by the photomultiplier tube for each pixel of a scanning matrix 128 × 128 or 128 × 64 with the pixel size 200 *μ*m. The fiber-optic probe (P/N:BF0060, Spectraconn Inc, Rock-away, New Jersey, USA) for raster scanning has six pieces of hexagonally close-packed UV-transparent quartz fibers transmitting excitation light, centered by one piece of quartz fiber transmitting emission light. All fibers have an inner diameter of 100 *μ*m. The distance between adjacent fiber rims is 40 *μ*m.

The raw imaging data of the NADH and Fp signals were further processed using a customized MATLAB^®^ program. A region of interest was carefully drawn along the tissue boundary to exclude the skin for each section. The nominal concentrations of both analytes were calculated by comparing the fluorescence intensity of the tissue to that of the reference standards of known concentrations. The concentration-based redox indices (NADH, Fp, Fp redox ratio, i.e. Fp/(NADH + Fp), and NADH/Fp) were used for the statistical analyses. Although NADH/Fp redox ratio and the Fp redox ratio are just two different formulas for the same redox state, their images are good for showing different features of tumor metabolic heterogeneity.

The mean value and the standard deviation (SD) of the redox indices of each tissue section were first computed. For global average analysis, these data were further averaged across sections for each individual tumor to obtain the tumor mean and SD, which were further averaged across tumors to get the mean values and the SDs of the tumor line. This analysis ignores the variations of the redox indices at different tissue depth. Student’s *t*-test was employed to investigate the statistical significance of each redox index and its SD between different groups.

To include the variations of the redox indices along tissue depth, the section-average-based univariate analysis model (General Linear Model, IBM SPSS statistics 20) was also used and is referred to as the section univariate analysis in this paper. The redox indices and their SDs were averaged for each image section and were then processed by the univariate analysis, where each redox index and its SD were set as the dependent variable, and either p53 status (for between groups) or tumor size group (for within p53 deletion group) as the independent or fixed factor, and tissue depth as a covariate. The section univariate analysis can detect the effect of the independent factor on tissue redox indices while controlling tissue depth of each section.

To quantitatively describe the heterogeneity pattern of the bimodal distribution of the redox state, two Gaussian functions were fitted to the histograms of the Fp redox ratio to obtain the mean values of the more oxidized state (higher Fp redox ratio, the corresponding region being defined as the core) and more reduced state (lower Fp redox ratio, the corresponding region being defined as the rim). The univariate analysis model was then applied similarly as aforementioned to either the core or rim averages. This method is referred to as the core–rim univariate analysis in this paper.

Only the redox indices with statistical significance (*p* < 0.05) were reported in all tables.

### 2.3. Histology

To understand the histological basis of the redox images, we investigated the H&E staining on the tumor tissue section (~5 *μ*m thick) adjacent to the last section optically scanned for a couple of tumors from each group. H&E staining was carried out by the Histology Core Facility of the Abramson Cancer Center at the Perelman School of Medicine, University of Pennsylvania. We took multiple photos (4X) of the H&E staining using a camera-equipped microscope (Olympus) and photo-stitched them using the photomerge function in Adobe^®^ Photoshop CS6 to show the image of the whole tissue section. No photo alteration was done except that the tissue sections were selected for presentation using the lasso tool in the Photoshop CS6. The H&E images were grossly compared to the redox images to identify the correlation of image features.

## 3. Results

**Fig. 1:**
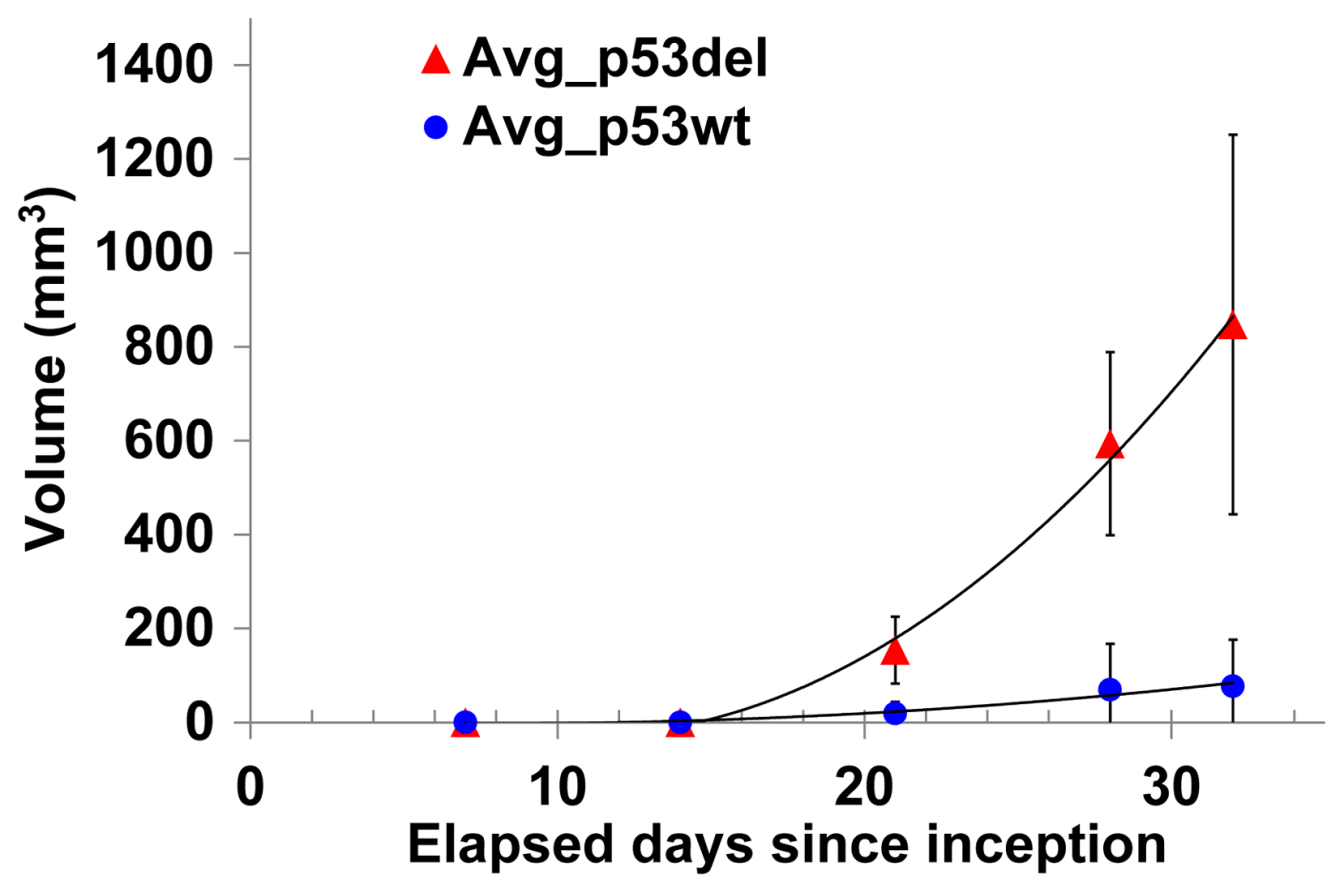
Tumor growth curve.

As a tumor suppressor gene, p53 inhibits the growth rate of HCT116 tumors, which we confirmed. After inoculation, about 60% mice grew p53 *wt* tumors and all mice grew p53^−/−^ tumors. Figure 1 is the growth curve of a batch of mouse xenografts. For the same batch, the p53^−/−^ tumors reached an average size 1260 × 550 mm^3^ (*N* = 7), over three times the average size of p53 *wt* group (364 ± 330 mm^3^, *N* = 3) 32 days after inoculation (*p* = 0.02). That the p53^−/−^ group grows significantly faster than the p53 *wt* group is consistent with the literature.^13^ Because the p53 deletion group grew faster than the p53 *wt* group, we divided the p53^−/−^ group into two subgroups: p53^−/−^ *small* (*N* = 3, vol = 760 ± 284 mm^3^) and p53^−/−^ *large* (*N* = 4, vol = 1634 ± 339 mm^3^). The tumor size of p53^−/−^ *small* is significantly smaller than that of the p53^−/−^ *large* as confirmed by the *t*-test (*p* = 0.015). The tumor size of the p53 *wt* group was matched up with the p53^−/−^ *small* group (*p* = 0.19).

### 3.1. Redox imaging identifies significant degree of redox state heterogeneity in all groups

In general, high degree of intratumor heterogeneity was observed for all tumors under investigation, regardless of their p53 status. Almost all tumors have an obvious bi-modal distribution of their redox state as represented by the Fp redox ratio. Some tumors have more oxidized central regions, some have more reduced central regions, while the others have “hot/cold” (oxidation/reduction) patchy redox state distribution patterns.

**Fig. 2:**
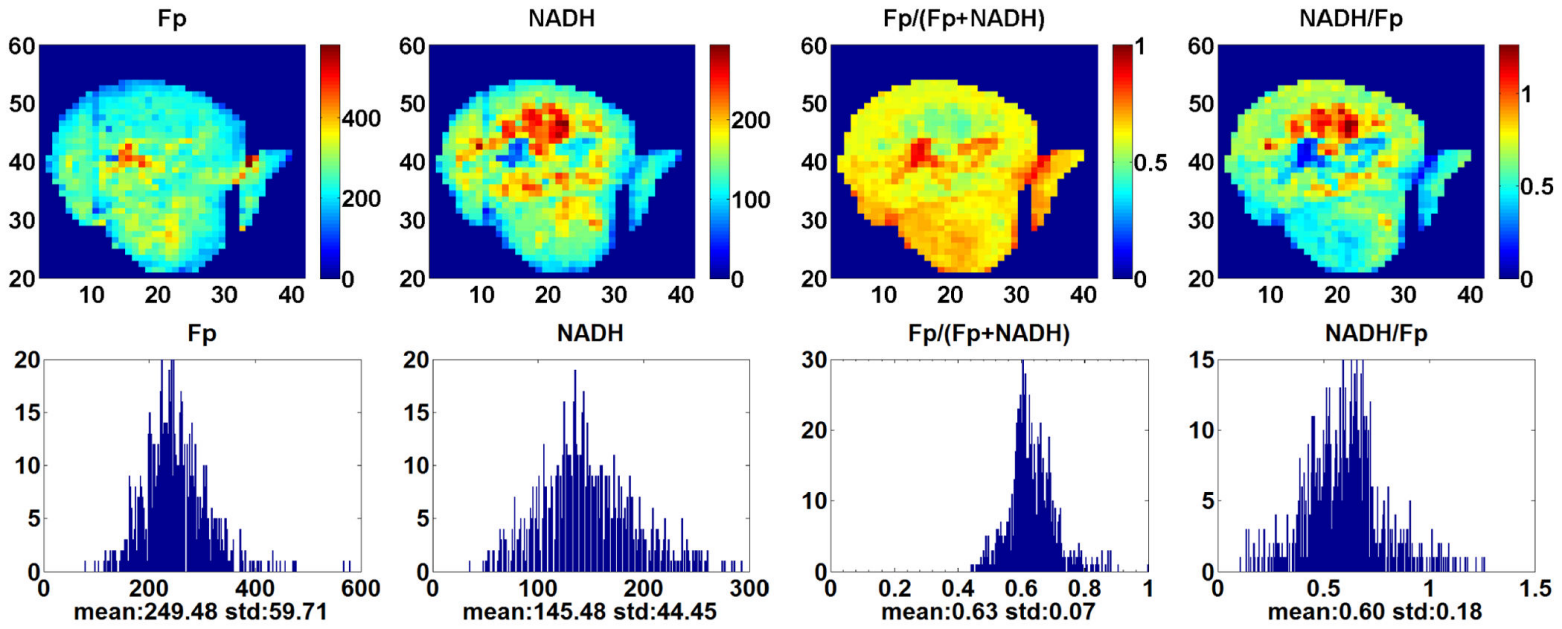
Typical redox images and corresponding histograms of the p53 *wt* group. The *x* and *y* axes represent the scanning matrix and the color bar is a reference to the colors in the corresponding image. Both Fp and NADH are in the unit of *μ*M in reference to the corresponding solution standard of the analytes. The spatial resolution is 200 *μ*m and the section depth is 1300 *μ*m.

**Fig. 3:**
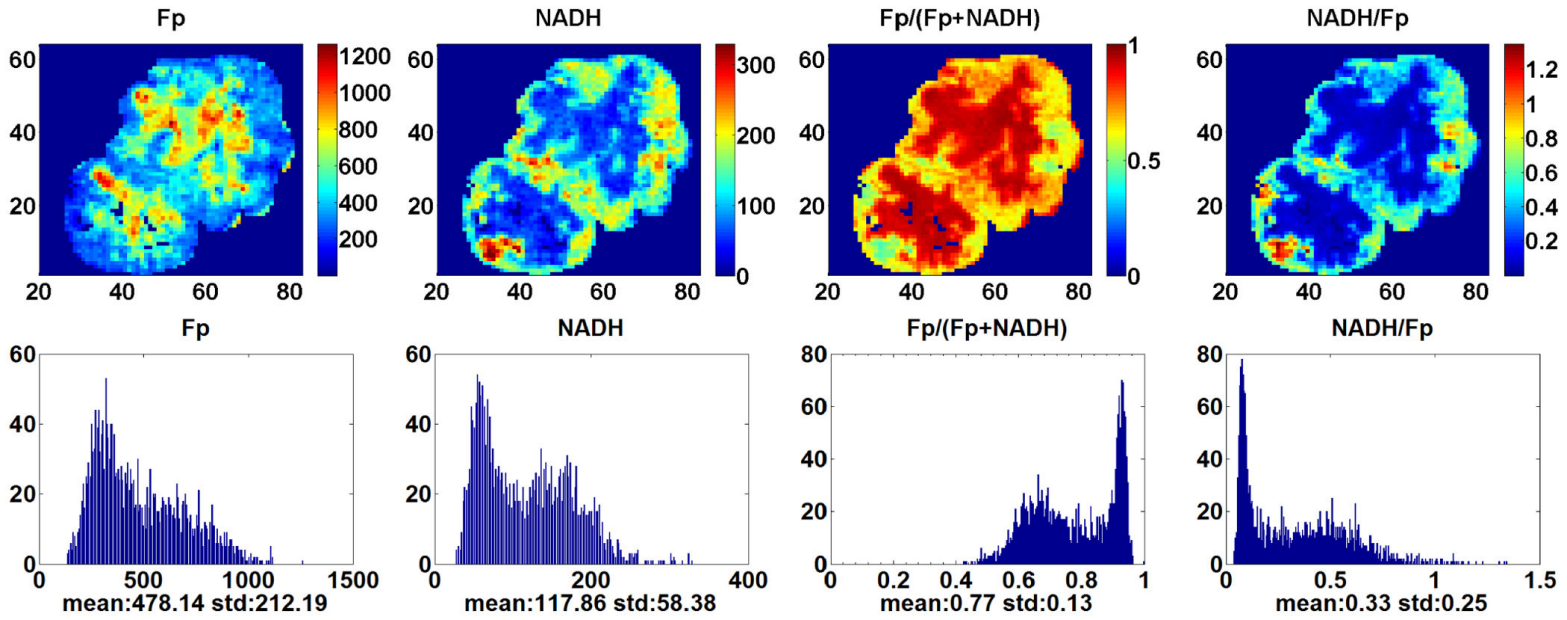
Typical images and corresponding histograms of the smallest tumor in the p53^−/−^ group. The spatial resolution is 200 *μ*m and the section depth is 2700 *μ*m.

**Fig. 4:**
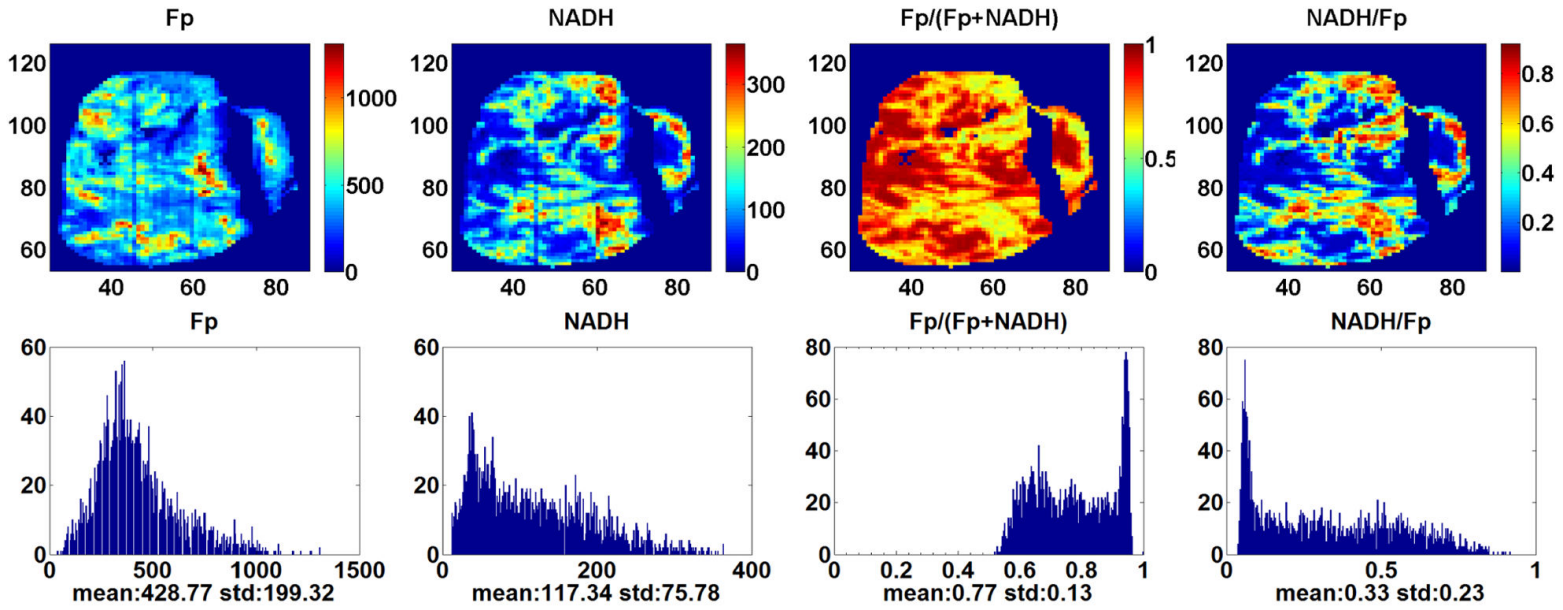
Typical redox images and corresponding histograms of the largest tumor in the p53^−/−^ group. The spatial resolution is 200 *μ*m and the section depth is 1400 *μ*m. The tumor size looks similar as the tumor in Fig. 3 because of the difference of section depth (The image size gets larger at deeper section depth).

Figure 2 displays the typical redox images of a tumor from the p53 *wt* group. The deep section of this tumor has reduced central region with oxidized hot patches. It is unusual to see a more reduced central region based on our previous tumor xenograft studies. Another tumor in the same group has just the opposite: oxidized central region and reduced periphery. Figure 3 shows the typical images of a tumor in the p53^−/−^*small* group. The central region is more oxidized. Figure 4 displays the typical images of a tumor in the p53^−/−^*large* group. The redox state distribution does not have any obvious oxidized central region but rather appears as a pattern of “hot” (more oxidized and higher Fp redox ratio) patches. Other tumors in the p53^−/−^ group exhibited either the core–rim pattern as in Fig. 3 or the “hot” oxidized patches as in Fig. 4.

### 3.2. The p53 null tumors are more oxidized and heterogeneous than the p53 wt ones

We compared the p53 *wt* group with the size matched p53^−/−^*small* group to evaluate their differences in the redox indices. With the global average analysis approach, we did not find significant difference in any of the redox indices. However, the redox ratio distribution was more heterogeneous in the p53^−/−^ group as the SD of the Fp redox ratio is larger than that of the wild type (*p* = 0.05), indicating the p53 null tumors have significantly higher degree of heterogeneity of the Fp redox state than the wild type (see Table 1).

On the basis of the section univariate analysis (see Sec. 2), Table 2 shows that the p53-deleted tumors had significantly less NADH (*p* = 0.004) and were more heterogeneous in the redox ratios (*p* = 2.6E-4 and 0.04 for the SD of the Fp redox ratio and NADH/Fp, respectively).

The difference between the p53^−/−^ and p53 *wt* groups is more distinctly recognized by performing the core–rim univariate analysis (see Sec. 2). All of the redox images of p53 null tumors show distinct patterns of intratumor heterogeneity and have a bimodal (core–rim) distribution in the histogram of the Fp redox ratio. To quantify the intratumor heterogeneity, we used two Gaussian functions to fit the Fp redox ratio histograms and obtained the mean values of the Fp redox ratio in the core and the rim. As shown in Table 3, we found that the p53^−/−^ small group is significantly more oxidized in the core (Fp redox ratio = 0.90 ± 0.03) than the p53 *wt* group in the core (Fp redox ratio = 0.82 ± 0.14) with a *p* value of 0.026, whereas we did not see significant difference in the Fp redox ratio of the rim between the two groups. The difference of the Fp redox ratio between the core and rim is significantly different (*p* = 0.001) between the two groups, indicating an increased polarization of the tissue redox state in p53 null tumors.

### 3.3. The size dependence of the tumor redox state is significant in the p53^−/−^ group

We further analyzed the data of the p53^−/−^ *small and* p53^−/−^ *large subgroups*. Table 4 summarizes the results of the section univariate analysis. We see that the p53^−/−^ *large* tumors have more Fp (*p* = 0.003) and are more oxidized (*p* = 0.034). In addition, we also see that they are significantly more heterogeneous in Fp measured by their larger SDs in Fp.

The core–rim univariate analysis shows that the p53^−/−^ *large* tumors are more oxidized in the core than the p53^−/−^ *small* tumors. Table 5 shows that the core of the p53^−/−^ *large* tumors is significantly more oxidized (Fp redox ratio = 0.92 ± 0.03) than that of the p53^−/−^ *small* ones (Fp redox ratio = 0.91 ± 0.03).

### 3.4. H&E staining images approximately correlate with the redox scanning images

**Fig. 5:**
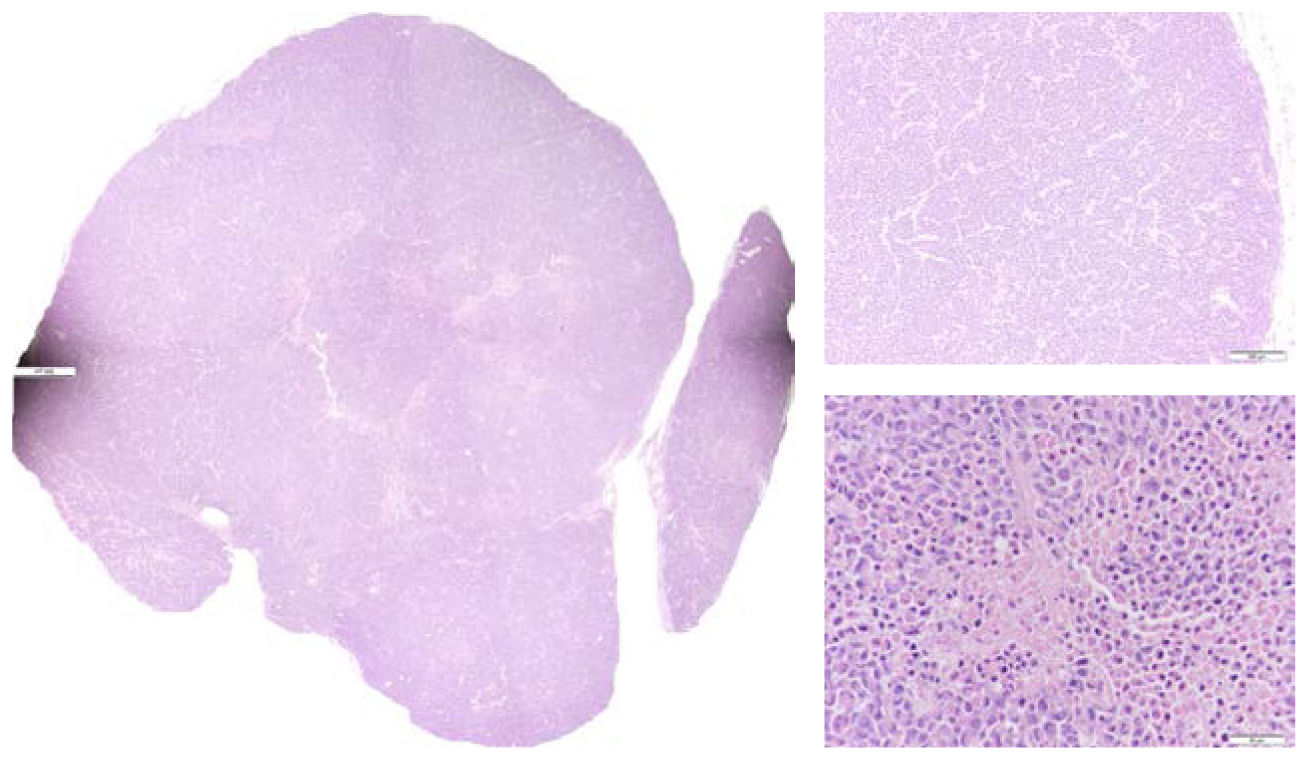
H&E staining for the p53 *wt* tumor in Fig. 2. Photo-stitched whole tissue section (L), typical tumor rim (top right, 10X) and typical tumor center (bottom right, 40X).

**Fig. 6:**
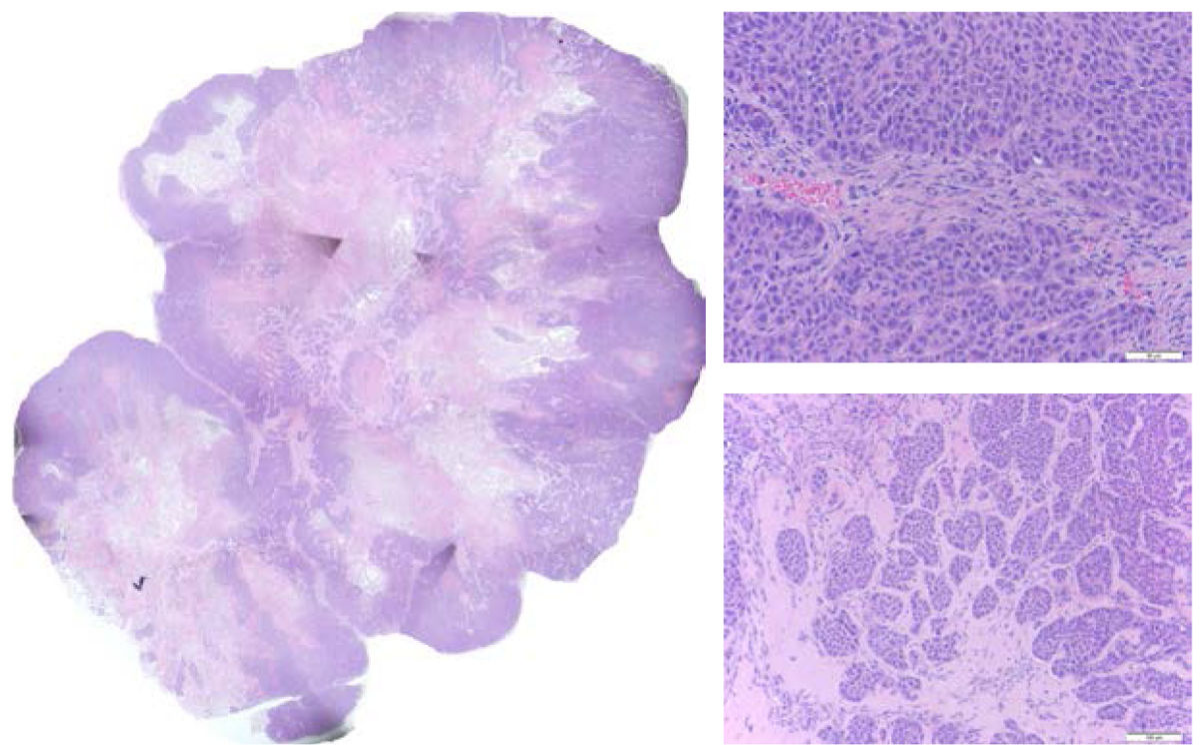
H&E staining for the p53^−/−^ tumor in Fig. 3. Photo-stitched whole tissue section (L), typical tumor rim (top right, 40X) and typical tumor center (bottom right, 20X).

**Fig. 7:**
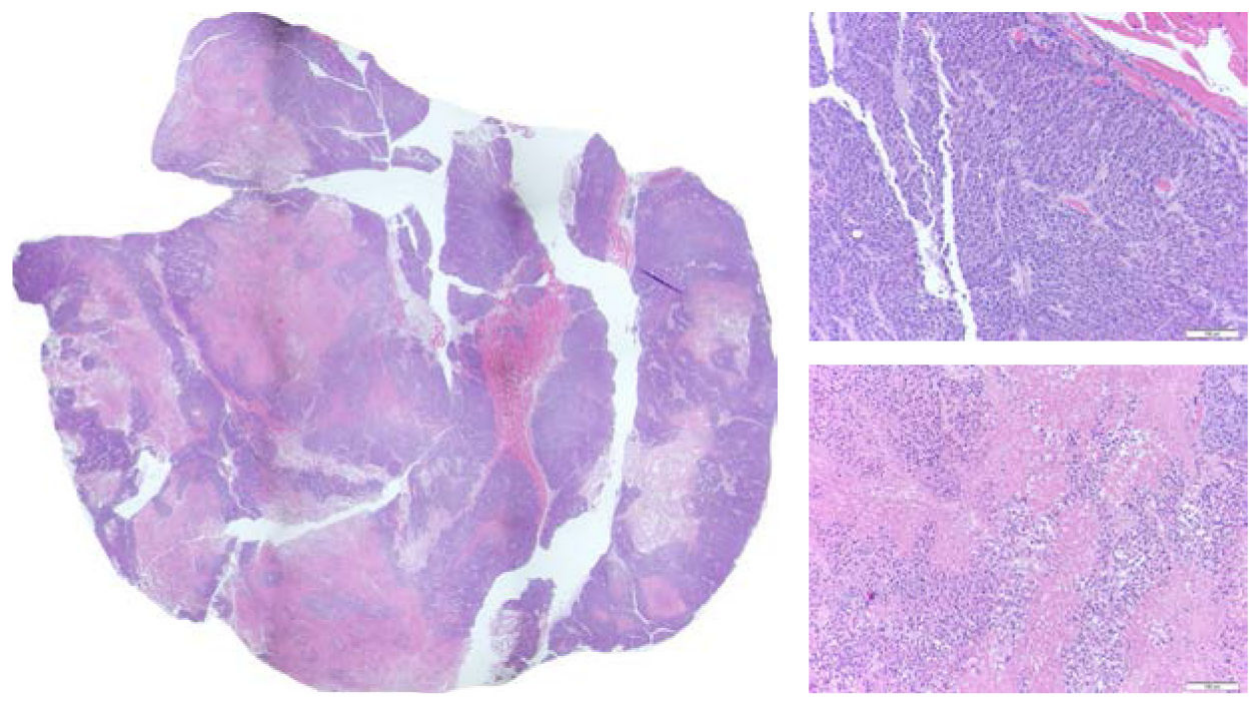
H&E staining for the p53^−/−^ tumor in Fig. 4. Photo-stitched whole tissue section (L), typical tumor rim (top right, 20X) and typical tumor center (bottom right, 20X).

Figures 5–7 are the H&E staining results corresponding to Figs. 2–4, respectively. Comparing the redox images with the H&E staining images, we can see that their patterns approximately correlate with each other. The NADH images positively correlate with the hematoxylin-stained viable cells and the Fp images correlate more with eosin-staining dominate patterns. The tumor shown in Figs. 2 and 5 only had a few necrotic cells in the central region evidenced in Fig. 5 (bottom right) with most portions being viable. This tumor had stronger NADH signals in the central region resulting in a reduced core. Although the H&E staining of the other two tumors shown in Figs. 3, 4, 6 and 7 showed apparent necrotic centers (reddish), islands of viable cells do exist within these areas and provided redox signals that have been detected.

## 4. Discussion

Intratumor heterogeneity has long been recognized as one of the characteristics of many forms of cancer, including colon cancer. For example, it was reported that two different subpopulations of colon carcinoma cells were originated from a single primary colon carcinoma.^14,15^ Also, colon carcinoma heterogeneity at cellular level has been characterized for developing more effective treatment.^16^ The high spatial resolution of the redox scanning technique clearly revealed the intratumor metabolic heterogeneity of the colon tumors that can be observed visually. HCT116 cells are known to be metastatic *in vivo*,^17^ and the typical oxidization-reduction pattern observed in these mouse xenografts is consistent with our previous observations on the mouse xenografts of metastatic melanoma and breast cancer.^5–7^

However, to quantitatively characterize the intratumor heterogeneity, proper statistical method should be used. The global average method minimized the degree of heterogeneity and failed to identify the significant differences in the redox indices between the p53 null and *wt* groups. Only the SD of the Fp redox ratio has a borderline significance between the two groups (see Table 1). The section univariate analysis addressed the heterogeneity with more details compared to the global average method. However, the heterogeneity within each tissue section is not fully taken into consideration by this method. The difference of NADH and SD of the Fp redox ratio were found to be highly significant between the two groups (see Table 2), while other indices were still not significantly different. The core–rim univariate analysis characterized the heterogeneity within each tissue section via the differentiation between the oxidized core and the reduced rim, which quantitatively shows the significant difference of the Fp redox ratio between the p53^−/−^ and p53 *wt* groups (see Table 3). In comparison, no statistical significance was found between the two groups when using core–rim global average analysis (data not shown). These results indicate that it is the tumor heterogeneity observed by the sufficient spatial resolution imaging that enables us to detect the possible connection between the redox state and the p53 status. The metabolic imaging approach as achieved by the Chance redox scanner facilitated us to uncover the dependence of the mitochondrial redox state on p53 status which might have been undetectable if using the conventional approaches sampling the whole tumor volume. It was reported that no significant difference was detected in glucose uptake between p53 *wt* and p53-null tumors using ^18^F fluoro-2-deoxyglucose positron emission tomography.^18^ Averaging over the whole tumor volume was used for this comparison. Likewise, when using the global averaging, we did not detect significant difference in any of the redox indices between the two groups, either. However, when the tumor core–rim heterogeneity was taken into account, statistically significant difference was identified in the present study. Our previous studies on mouse models of melanoma,^7^ breast cancer^6^ and pancreatic premalignancy^19^ demonstrated the successes of the same approach. It was the redox indices characterizing tumor metabolic heterogeneity rather than the tumor global averages that identified the difference among tumors of different aggressiveness. Thus, it appears that imaging tumor metabolism at sub-millimeter resolution is the key, since it allows us to characterize the heterogeneity features of metabolism. In future, we may investigate glucose uptake of these tumors using the Chance redox scanner to simultaneously image the redox indices and Pyro-2DG (a 2-deoxyglucose analog with near infrared fluorescence) at submillimeter resolution.^20^

The p53 dependence of the redox state in these colon cancer xenografts suggests that alteration in p53 tumor suppressor gene leads to profound metabolic changes in tumor, such as the mitochondrial redox state, and p53 may play a critical role in regulating the mitochondrial metabolism. These findings also support the previous reports showing that p53 regulates metabolic activity through induction of TIGAR (T P53-induced inhibitor of glycolysis and apoptosis regulator) and SCO2.^3,21^ It is known that p53 mutation suppresses mitochondrial respiration and enhances glycolysis, which could result in more oxidized mitochondrial redox state as we observed.

Considering the complexity and heterogeneity of tumors *in vivo* compared to the cells cultured *in vitro*, more characterization of the signaling and metabolic pathways using appropriate imaging methods should aid our understanding the mechanisms of p53 dependence of the redox state. For example, it would be interesting to probe whether the p53 status is the same between the oxidized core and reduced rim, and use multi-modality imaging with sufficient spatial resolution to detect some other aspects of tumor metabolism such as glycolysis, oxygen consumption and adenosine triphosphate (ATP) generation and how they may interplay with the p53 pathways. Tumor size is another factor that should be considered which may well be related to the tumor blood supply, metabolism, and micro environment. We do not have data about the tumor size dependence of the redox state in the p53 *wt* group, which can be studied in the future.

The underlying histological basis for the oxidized/ reduced redox state is still unclear. The core–rim (oxidation-reduction) patterns revealed by the redox images, usually with the oxidized core located in the central region and the reduced rim located in the peripherals have been demonstrated in our prior studies in mouse xenografts of metastatic cell lines of human melanoma and breast cancer.^6,7^ The oxidized cores were observed even when the tumor size was as small as 4–6 mm with no apparent sign of necrosis, and some non-invasive NMR imaging results on the tumors also showed that the oxidized cores cannot be explained just by necrotic centers.^22^ Viable cells with intact nuclei were observed in the cores of the melanoma xenografts.^23^ The simultaneous imaging of the glucose uptake and the mitochondrial redox state and the histological studies on the large mouse xenografts of breast cancer indicated that there were also viable cells existing in the oxidized areas which are commonly regarded as necrotic centers.^24^ Together, our redox scanning studies on melanoma, breast cancer, and colon cancer xenografts demonstrate that the oxidized cores may provide important clues to tumor metastatic potential. It would be interesting to study in more detail the biological difference between the cells in the core and rim in these colon cancer xenografts.

## 5. Conclusions

We reported the 3D redox imaging data of the colon tumor lines with p53 null and p53 *wt* status in mouse models. To the best of our knowledge, the present work is the first to reveal the intratumor metabolic heterogeneity pattern of the redox state in colon cancer and the first to show the possible p53-dependence of the mitochondrial redox state at tissue level with submillimeter spatial resolution. The findings should assist in our understanding on colon cancer pathology and developing new imaging biomarkers for clinical applications.

## Figures and Tables

**Table 1:** Global average analysis results of size matched p53^−/−^ and p53 *wt* tumors.

p53 status	Vol (mm^3^)	SD_Fp redox ratio
p53*wt* (*N* = 3)	364 ± 330	0.07 ± 0.01
p53^−/−^*small* (*N* = 3)	760 ± 284	0.11 ± 0.02
*p*	0.19	0.05

**Table 2:** Section univariate analysis results of size matched p53^−/−^ and p53 *wt* tumors.

p53 status	NADH (*μ*M)	SD–Fp redox ratio	SD_NADH/Fp
p53 *wt* (*N* = 3, *S* = 12)	100 ± 22	0.07 ± 0.01	0.14 ± 0.03
p53^−/−^ *small* (*N* = 3, *S* = 15)	78 ± 31	0.11 ± 0.02	0.18 ± 0.05
*p*	0.004	2.6E-04	0.04

**Table 3:** The differences between the p53 *wt* and p53^−/−^ *small* groups via the core–rim univariate analysis.

p53 status	Fp Redox ratio_core	ΔFp Redox ratio (core–rim)
p53 *wt* (*N* = 3, *S* = 11)^a^	0.82 ± 0.14	0.12 ± 0.04
p53^−/−^ small (*N* = 3, *S* = 15)	0.90 ± 0.03	0.19 ± 0.04
*p*	0.026	0.001

a A top section was excluded due to its homogeneity.

**Table 4:** Section univariate analysis results on the size dependence of the redox indices within the p53^−/−^group.

p53 deletion	Vol (mm^3^)	Fp (*μ*M)	Fp Redox ratio	SD–Fp (uM)
p53^−/−^ large (*N* = 4, *S* = 14)	1634 ± 339	411 ± 83	0.82 ± 0.06	192 ± 35
p53^−/−^ small (*N* = 3, *S* = 15)	760 ± 284	343 ± 119	0.79 ± 0.03	166 ± 55
*p*	0.015 (*t*-test)	0.003	0.034	0.008

**Table 5:** Core–rim univariate analysis on the size dependence of redox indices within the p53^−/−^ group.

p53 deletion	Fp Redox ratio core
Large (*N* = 4, *S* = 14)	0.92 ±0.03
Small (*N* = 3, *S* = 15)	0.91 ±0.03
*p*	0.047
